# IC100: a novel anti-ASC monoclonal antibody improves functional outcomes in an animal model of multiple sclerosis

**DOI:** 10.1186/s12974-020-01826-0

**Published:** 2020-05-04

**Authors:** Haritha L. Desu, Melanie Plastini, Placido Illiano, Helen M. Bramlett, W. Dalton Dietrich, Juan Pablo de Rivero Vaccari, Roberta Brambilla, Robert W. Keane

**Affiliations:** 1 University of Miami Miller School of Medicine, Miami, FL 33136 USA; 2 InflamaCORE, LLC, Miami, FL 33156 USA; 3 Bruce W. Carter, Department of Veterans Affairs Medical Center, Miami, FL 33136 USA; 4 Deparment of Neurobiology Research, Institute of Molecular Medicine, University of Southern Denmark, Odense, Denmark; 5 BRIDGE Brain Research Inter Disciplinary Guided Excellence, Department of Clinical Research, University of Southern Denmark, Odense, Denmark; 6 Department of Physiology and Biophysics, University of Miami Miller School of Medicine, Miami, FL 33136 USA

**Keywords:** IC100, Inflammasome, Neuroinflammation, Multiple sclerosis, Experimental autoimmune encephalomyelitis, Caspase-1, IL-1, ASC, Pycard

## Abstract

**Background:**

The inflammasome adaptor apoptosis-associated speck-like protein containing a CARD (ASC) is involved in immune signaling by bridging the interactions between inflammasome sensors and caspase-1. Strong experimental evidence has shown that ASC^−/−^ mice are protected from disease progression in animal models of multiple sclerosis (MS), suggesting that targeting inflammasome activation via ASC inhibition may be a promising therapeutic strategy in MS. Thus, the goal of our study is to test the efficacy of IC100, a novel humanized antibody targeting ASC, in preventing and/or suppressing disease in the experimental autoimmune encephalomyelitis (EAE) model of MS.

**Methods:**

We employed the EAE model of MS where disease was induced by immunization of C57BL/6 mice with myelin oligodendrocyte glycoprotein peptide 35–55 (MOG_35–55_). Mice were treated with vehicle or increasing doses of IC100 (10, 30, and 45 mg/kg) and clinical disease course was evaluated up to 35 days post EAE induction. Immune cell infiltration into the spinal cord and microglia responses were assessed.

**Results:**

We show that IC100 treatment reduced the severity of EAE when compared to vehicle-treated controls. At a dose of 30 mg/kg, IC100 significantly reduced the number of CD4^+^ and CD8^+^ T cells and CD11b^+^MHCII^+^ activated myeloid cells entering the spinal cord from the periphery, and reduced the number of total and activated microglia.

**Conclusions:**

These data indicate that IC100 suppresses the immune-inflammatory response that drives EAE development and progression, thereby identifying ASC as a promising target for the treatment of MS as well as other neurological diseases with a neuroinflammatory component.

## Background

Multiple sclerosis (MS) is an autoimmune demyelinating disease of the central nervous system (CNS) characterized by an inflammatory response sustained by innate and adaptive immune mechanisms dependent on lymphocyte (both T and B cells) and myeloid cell activation [1]. The direct immune attack on myelin-producing oligodendroglia causes demyelination, leading to axonal and neuronal damage that correlates with long-term disability [2]. Approximately 85% of MS patients present with a relapsing-remitting phenotype (RRMS), and the majority of these evolve into a secondary-progressive (SPMS) disease course after 15 to 20 years. Approximately 10–15% of patients experience a primary progressive (PPMS) disease course with slow and continuous deterioration without definable relapses [3]. The understanding of MS pathomechanisms as well as the development of MS therapeutics has relied upon the use of the experimental autoimmune encephalomyelitis (EAE) model. In fact, many of the disease-modifying drugs currently in clinical use have been developed after successful testing in EAE [4].

Inflammasomes are the central signaling platforms of the innate immune-inflammatory response. Upon activation by a diverse range of intracellular sensors of infection, cell damage or metabolic disturbances, the adaptor protein apoptosis speck-like staining protein containing a caspase recruitment domain (ASC), common to all inflammasomes, is recruited. Sensor oligomerization induces the polymerization of ASC into highly cross-linked macromolecular assemblies called ASC specks or pyroptosomes [5]. ASC recruits pro-caspase-1, resulting in proximity-induced proteolytic cleavage of pro-caspase-1. Mature caspase-1 processes the pro-inflammatory cytokines interleukin-(IL)-1β and IL-18, as well as a range of other molecules. ASC specks are released by inflammasome-activated cells into the extracellular space and in body fluids where they continue to recruit and activate pro-caspase-1 and to process the maturation of IL-β [6, 7]. Extracellular specks are taken up by neighboring cells and function to propagate the innate immune response [8]. ASC specks have been detected in tissues and sera of patients and mice with autoimmune and inflammatory diseases [6, 7], and serum levels of ASC are increased in MS patients [9]. The model of transcellular propagation of inflammasome activation via ASC specks suggests that extracellular ASC might be vulnerable to antibody-mediated therapies. We and others have observed that passive immunization with anti-ASC antibodies reduces pathology in several mouse models of excessive inflammasome activation [10–14]. In support of this idea is the finding that anti-Tau antibodies promote uptake of Tau aggregates into microglia and that these effects are epitope and aggregate size-dependent [15]. These studies therefore identify microglia as potential important effectors of therapeutic antibodies and demonstrate that anti-Tau antibodies are taken up by microglia and facilitate the clearance of extracellular Tau [15].

Emerging evidence has indicated important inflammasome-independent roles for ASC in controlling adaptive immune responses [16–19]. ASC regulates adaptive immunity by modulating Dock-2-dependent Rac activation and actin polymerization in dendritic cells and lymphocytes thereby regulating the motility of T lymphocytes and B lymphocytes and antigen uptake by professional antigen-presenting cells [17]. Thus, the vital role of ASC in regulating both adaptive and innate immune responses suggests that strategies to modulate ASC expression appear to be promising approaches for the treatment of CNS inflammatory diseases.

The NLRP3 and NLRC4 inflammasome have been previously associated with the pathology of MS [20, 21]. The NLRP3 inflammasome appears critical in the development of EAE, as demonstrated by the resistance of *Nlrp3*^*−*/*−*^ mice to EAE [22, 23]. The lack of the NLRP3 inflammasome in antigen-presenting cells dampens expression of chemokines and chemokine receptors on T helper cells and antigen-presenting cells, and prevents their migration to the CNS [23]. Alternatively, gene deletion of NLRP3 inflammasome components in mice subjected to EAE showed that progression of EAE is dependent on the inflammasome adaptor protein ASC and caspase-1 but not NLRP3 [19]. ASC^−/−^ mice were protected from EAE more than caspase-1^−/−^ mice, suggesting that an inflammasome-independent function of ASC contributes to EAE progression. The deficiency in ASC did not affect MOG-specific T cell proliferation or cytokine production in the periphery. However, ASC appeared to play a role in the peripheral survival of mature CD4^+^ T cells [19]. ASC^−/−^ mice showed reduced numbers of MOG-specific T cells in the lymph nodes and in the CNS, resulting in protection from EAE. To explain these discrepancies, it has been suggested that EAE induced by weak activation of innate immunity requires the NLRP3 inflammasome, whereas strong activation of innate immunity makes the EAE pathological mechanism bypass the NLRP3 inflammasome and causing EAE to develop without the NLRP3 inflammasome [23].

Monoclonal antibodies (mAb) have been effectively used in MS therapy [24]. One of their main advantages is that, due to their high target specificity, they have minimal unwanted side effects. Natalizumab, which targets α4ß1 integrin, and alemtuzumab, directed against the lymphocyte surface marker CD52, are among the most effective mAb currently in clinical use [24]. Recently, ocrelizumab, targeting the B cell antigen CD20, has been the first drug approved by the US Food and Drug Administration (FDA) for PPMS [25–27]. Given the proven involvement of the inflammasome in MS and EAE pathobiology, we sought to pursue the testing of a mAb-targeting ASC and ASC specks to improve clinical outcomes in EAE and MS.

In this study, we evaluated the therapeutic efficacy of a humanized monoclonal antibody against human ASC, IC100, in MOG-induced EAE. We show that IC100 significantly suppressed disease severity when compared to vehicle-treated controls. At a dose of 30 mg/kg, IC100 reduced the number of CD4^+^ and CD8^+^ T cells and CD11b^+^MHCII^+^ activated myeloid cells in the spinal cord. In parallel, it reduced the number of total and activated microglial cells in the spinal cord. These data indicate that IC100 suppresses the adaptive and innate immune-inflammatory response that drives EAE, thereby identifying ASC as a promising target for MS therapy.

## Methods

### Induction of EAE and development and treatment with IC-100

Active EAE was induced in 2-months old C57BL/6 female mice with myelin oligodendrocyte glycoprotein 35–55 peptide (MOG_35–55_, BioSynthesis) as previously described [28]. Briefly, mice received an intraperitoneal (i.p.) injection of pertussis toxin dissolved in PBS (350 ng/mouse; day 0), followed by sub-cutaneous administration of MOG_35–55_ (300 ng/mouse; day 1) emulsified in complete Freund’s adjuvant, and a second i.p. injection of pertussis toxin (350 ng/mouse; day 2). IC100 (IgG4) was developed by humanization of a mouse monoclonal (IgG1) against human ASC (Abzena, Cambridge England). IC100 was cloned into a CHO cell manufacturing cell line (Selexis, Geneva, Switzerland). IC100 was purified from CHO cell supernatants using ProSepA high capacity column chromatography (Antibody Solutions, Santa Clara, CA). Mice were administered vehicle (0.9% saline) or IC100 at three different doses (10, 30, and 45 mg/kg) via i.p. injection every 4 days, starting at day 8 after induction of EAE. Clinical symptoms of EAE were assessed daily by a blind investigator on a scale of 0 to 6 as follows: 0, no clinical signs; 1, loss of tail tone; 2, completely flaccid tail; 3, complete hind limb paralysis; 4, complete forelimb paralysis; 5, moribund; 6, dead. All experiments were performed according to protocols and guidelines approved by the Institutional Animal Care and Use Committee of the University of Miami.

### Cell isolation for flow cytometry

Following transcardial perfusion with PBS, spinal cords were harvested and placed in cold Hanks’ Balanced Salt Solution without Mg^2+^ and Ca^2+^ (HBSS w/o). Samples were then manually dissociated into single cell suspensions through a 70-μm strainer and washed in HBSS w/o. Spleen samples were spun at 1200 rpm for 10 min at 4 °C, supernatants were removed, and red blood cells (RBCs) were lysed in 2 ml RBC lysis buffer (eBioscience) according to the manufacturer’s instructions. Spleen cells were then resuspended in PBS. Cells isolated from the spinal cord were resuspended in flow cytometry buffer (FCB, eBioscience) and incubated with Myelin Removal Beads II (Miltenyi). Myelin was depleted using the LS magnetic columns as described in the manufacturer’s protocol (Miltenyi). Similar to the splenocytes, spinal cord cells were resuspended in PBS and stained as described below.

### Immunolabeling and flow cytometric analysis

Cells were resuspended in 100 μl FCB, blocked with 0.5 μl TruStainFcX (anti-mouse CD16/32 FcR block, Biolegend) for 5 min at room temperature, and stained for 30 min at 4 °C with: FITC-anti-CD45 (1:1000, Biolegend), PE-Cy7-anti-CD4 (1:200, eBioscience), PerCP-Cy5.5-anti-CD8 (1:200, Biolegend), PE-anti-B220 (1:200, Biolegend), APC-anti-MHCII (1:200, eBioscience), and APC-eFluor780-anti-CD11b (1:200, eBioscience) (Supplemental Table 1). Cells were then fixed in 1% PFA for 1 h. Finally, cells were resuspended in 500 μl FCB, labelled with DAPI to exclude cell debris, and analyzed with a CytoFLEX S flow cytometer (Beckman-Coulter) equipped with the CytExpert software (Beckman-Coulter).

### Quantification of IC100 in tissues

IC100 was quantified in the brain, spinal cord, liver, and spleen at 35 days post-induction (dpi) of EAE using a proprietary assay developed by InflamaCORE, LLC using Meso Scale Technology. Protein lysates were obtained as described in [29]. The assay was read using the QuickPlex SQ 120 instrument (Meso Scale Diagnostics, Maryland).

### Statistical analyses

EAE curves were analyzed point-by-point by 2-way repeated measures ANOVA, followed by Tukey test for multiple comparisons. The EAE curves as a whole were also analyzed by non-parametric Mann-Whitney test comparing treated groups to the control group, individually. All other assessments were analyzed by Student’s *t* test, if the data were normally distributed, or by non-parametric Mann-Whitney test if the data were not normally distributed. Normality was determined by the Pearson & D’Agostino test. Data were expressed as mean ± SEM, and *p* values equal or less than 0.05 were considered statistically significant. Statistical analyses were carried out with the GraphPad Prism software.

## Results

### Treatment with IC100 ameliorates the functional outcome in EAE

**Fig. 1:**
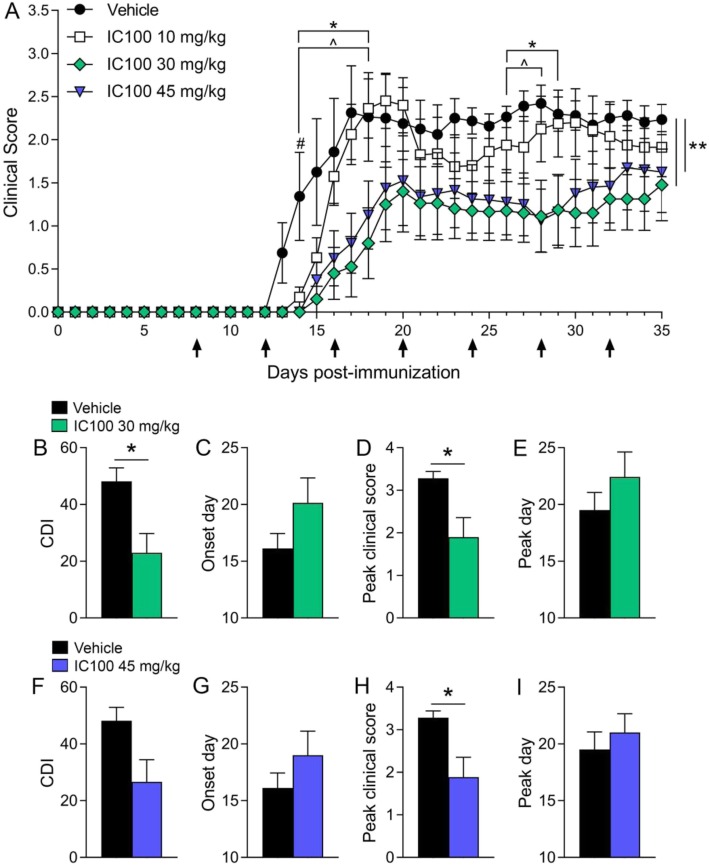
IC100 treatment improves the functional outcome in EAE. **a** Clinical course of MOG_35-55_-induced EAE in C57BL/6 mice treated with vehicle or increasing doses of IC100 (10, 30, and 45 mg/kg i.p. every 4 days) with administration starting at day 8. Results are expressed as daily mean clinical score ± SEM of 9–10 mice/group. Statistical analysis by 2-way repeated measures ANOVA with Tukey’s multiple comparisons test: The number sign indicates significantly different time point, Vehicle vs IC100 10 mg/kg: *p* value 14 dpi = 0.0313. The asterisk indicates significantly different time points, vehicle vs IC100 30 mg/kg: *p* values 14–18 dpi = 0.0089, 0.0031, 0.0053, 0.0002, 0.0034; *p* values 27–30 dpi = 0.0191, 0.0116, 0.046, 0.04. The caret symbol indicates significantly different time points, vehicle vs IC100 45 mg/kg: *p* values 14–18 dpi = 0.0089, 0.0179, 0.02, 0.0023, 0.0377; *p* values 27–29 dpi = 0.0377, 0.0079, 0.0392. The double asterisk symbol indicates curve comparisons: vehicle vs IC100 30 mg/kg, *p* = 0.0025; vehicle vs IC100 45 mg/kg, *p* = 0.004, Mann-Whitney test. **b**–**i** Comparison of EAE parameters between vehicle-treated mice and each of the two effective doses: **b**, **f** Cumulative Disease Index (CDI), calculated as sum of daily scores from day of onset for each animal (measure of EAE severity), *p ≤ 0.05, Mann-Whitney test; **c**, **g** Onset day, considered as day a mouse showed EAE symptoms for two consecutive days; (**d**, **h**) Peak clinical score, considered as highest score reached by a mouse acutely after onset, *p ≤ 0.05, Student’s *t* test; **e**, **i** Peak disease day, considered as day a mouse reached the highest disease score acutely after onset

In order to assess the therapeutic potential of IC100, we induced EAE with MOG_35–55_ peptide [30] in 2-months old female C57BL/6 mice and administered IC100 or vehicle (saline) alone beginning 8 days post-induction (dpi) of the disease. The administration was repeated every 4 days until sacrifice (35 dpi). Three doses were tested, 10, 30, and 45 mg/kg. IC100 significantly improved functional recovery when used at the doses of 30 and 45 mg/kg, with a robust reduction of the clinical disease scores throughout the duration of the experiment (Fig. 1a). The average peak clinical score was significantly reduced at both the 30 and 45 mg/kg doses (Fig. 1d, h), and the overall severity of EAE measured with the cumulative disease index (CDI) was reduced by half with the 30 mg/kg dose (Fig. 1c). Because the beneficial effect reached a plateau at 30 mg/kg, we cannot exclude that we may be able to lower the dose further or reduce the frequency of administration and obtain comparable or even enhanced benefits as the 30 mg/kg dose used here, while minimizing the chances of developing neutralizing antibodies against IC100 that may abrogate its effect.

### Treatment with IC100 reduces the infiltration of peripheral immune cells into the spinal cord following EAE

**Fig. 2:**
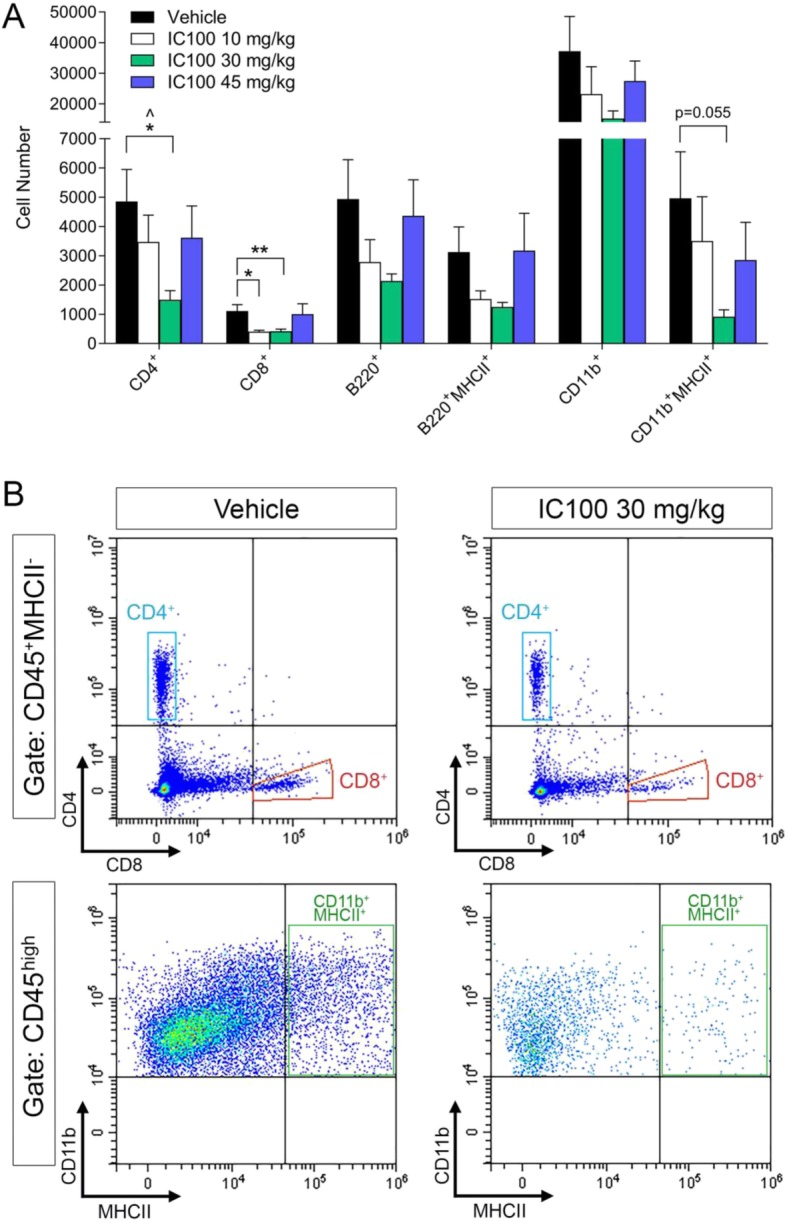
IC100 treatment reduces peripheral immune cell infiltration into the spinal cord following EAE. **a** Flow cytometric quantification of the leukocyte populations infiltrating into the spinal cord at 35 dpi after EAE. The flow cytometry gating strategy is provided in Supplementary Figure 1. **b** Representative flow cytometry plots of CD4 and CD8 T cell populations from mice treated with vehicle and IC100 (30 mg/kg). In order to assure that no dendritic cells expressing CD4 or CD8 were included, we identified the CD4^+^ and CD8^+^ T cell populations after gating on the CD45^+^MHCII^−^ population. Results are expressed as average ± SEM of 5 mice/group, *p < 0.05, **p < 0.001, Mann-Whitney *t* test; ^*p* < 0.05, one-way ANOVA, Dunnet’s multiple comparisons test

Initiation, persistence, and severity of the clinical symptoms in EAE are directly correlated with the infiltration of immune cells into the spinal cord. To evaluate whether IC100 altered this process, we analyzed by flow cytometry the profile of the immune cell populations isolated from the spinal cord at 35 dpi. Treatment with the effective dose of 30 mg/kg significantly reduced the total number of encephalitogenic CD4^+^ T cells as well as CD8^+^ T cells (Fig. 2a, b), the immune cell populations most crucial in driving EAE pathology. Surprisingly, the 45 mg/kg dose did not produce a reduction in spinal cord immune cell infiltration, despite reducing the overall severity of EAE. However, it is possible that at the high dose animals developed neutralizing antibodies against IC100 that diminished potency of the drug. On the other hand, it is important to note that immune cell infiltration was measured at 35 dpi, point beyond the acute phase of EAE, and it is possible that a second wave of immune activation contributes to the overall pathology. Indeed, this idea correlates with the mild elevation of the average clinical scores observed after 30 dpi in the 45 mg/kg treated group.

**Fig. 3:**
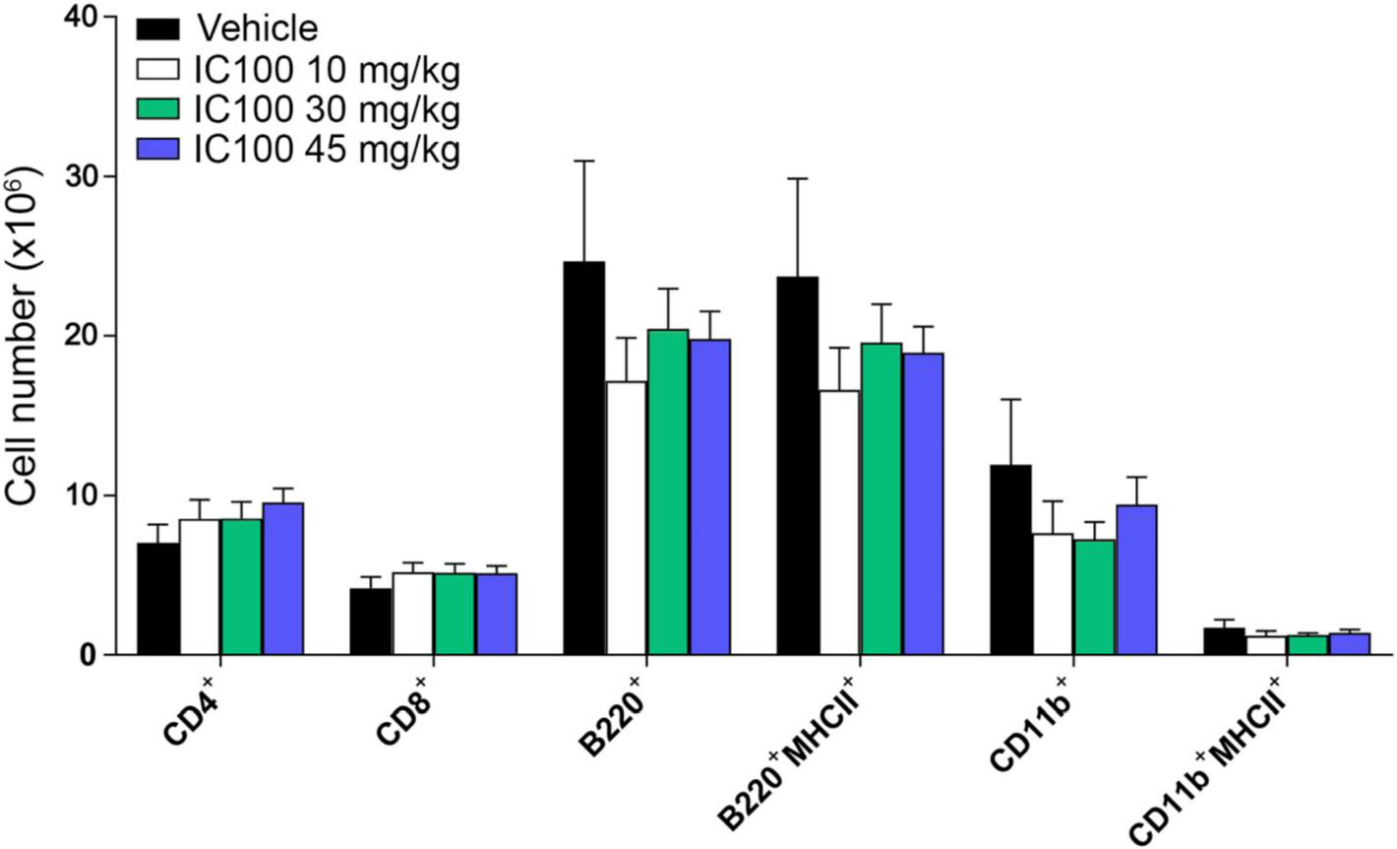
IC100 treatment does not affect splenic immune cell populations following EAE. Flow cytometric quantification of the leukocyte populations in the spleen at 35 dpi EAE. Results are expressed as average ± SEM of 5 mice/group

Except for the T cell population, the numbers of all other measured immune cell populations did not change, although a clear downward trend was observed particularly for CD11b^+^ myeloid cells (macrophages and neutrophils combined) (Fig. 2a, b). No differences in cell numbers were observed with any of the IC100 doses in the spleen, suggesting that treatment did not interfere with the ability of mice to mount an adequate immune response to the EAE challenge (Fig. 3). The gating strategy for the flow cytometry experiments is reported in Supplementary Figure 1.

### Treatment with IC100 reduces the number and activation state of microglia following EAE

**Fig. 4:**
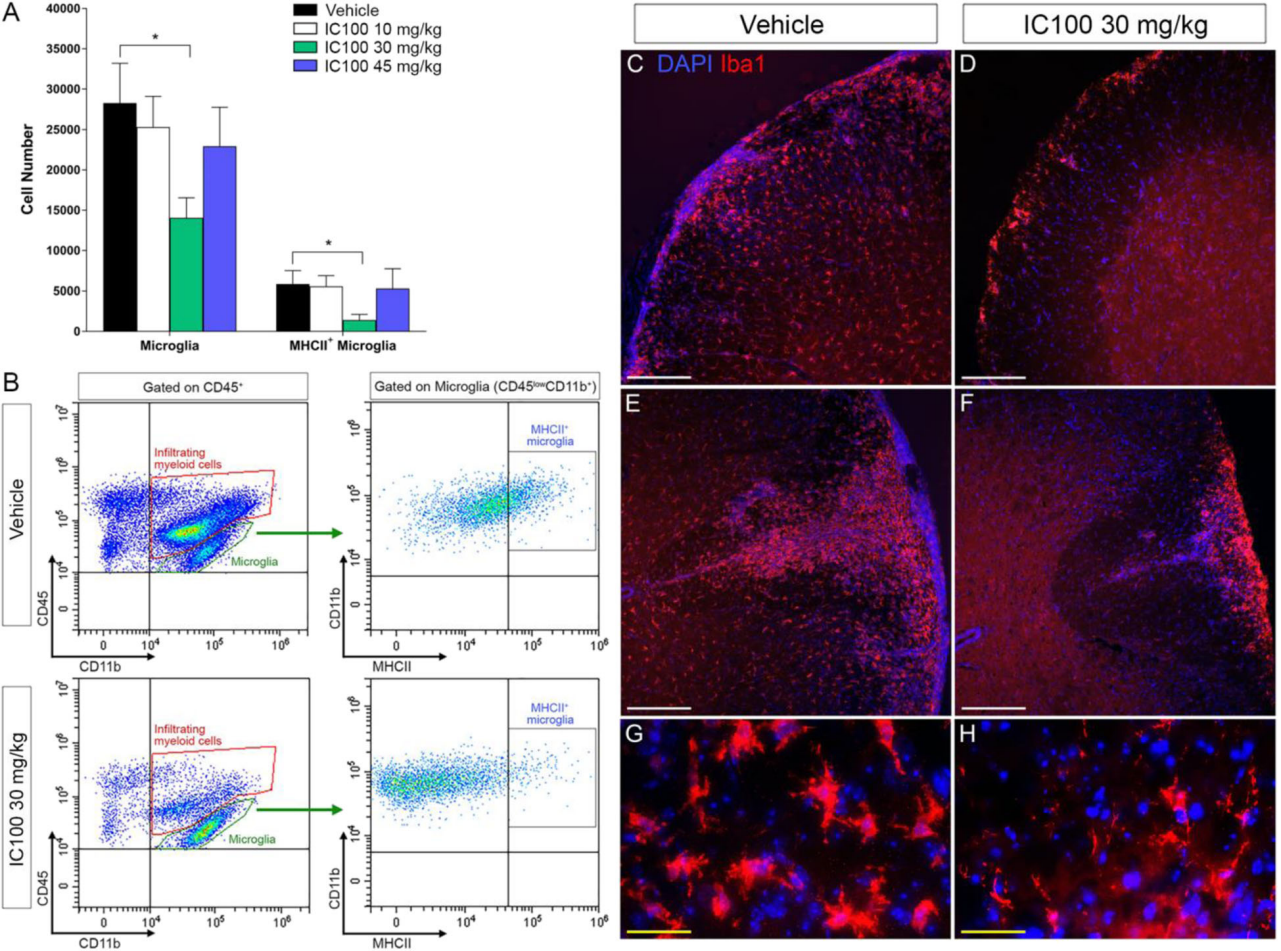
IC100 treatment reduces number and activation state of microglia following EAE. **a** Flow cytometric quantification of CD45^low^CD11b^+^ total microglia and CD45^low^CD11b^+^MHCII^+^ activated microglia in the spinal cord at 35 dpi after EAE. **b** Representative flow cytometry plots of CD45^low^CD11b^+^ total microglia (green circles) and CD45^low^CD11b^+^MHCII^+^ activated microglia (right panels) from mice treated with vehicle and IC100 (30 mg/kg). Results are expressed as average ± SEM of 5 mice/group, *p < 0.05, Student’s *t* test; no statistically significant differences were observed by one-way ANOVA with Dunnet’s multiple comparisons test. **c** Iba1 immunostaining of microglia and infiltrating myeloid cells in the spinal cord of mice treated with vehicle and IC100 (30 mg/kg). White scale bar = 200 μm; yellow scale bar = 50 μm

Microglia participate in the immune-inflammatory response associated with CNS disease. Their activation is characterized by increased proliferation and upregulation of MHCII surface expression. To assess whether IC100 increased microglial cell activation, we quantified the number of total microglia, identified as the CD45^low^CD11b^+^ population (Fig. 4b, green circles), and MHCII^+^ activated microglia (Fig. 4b, right panels) in the same spinal cord samples by flow cytometry. Total microglia were highly reduced by IC100 at 30 mg/kg, and so were the MHCII^+^ activated microglia (Fig. 4a). This finding was confirmed by Iba1 immunostaining of spinal cord tissue sections where vehicle-treated mice displayed a clear increase in microglia numbers throughout the white and gray matter compared to the 30 mg/kg IC100-treated group (Fig. 4c). In the vehicle-treated group, microglia also appeared more ramified and hypertrophic. Together, these data indicate that IC100 is effective in suppressing microglia activation and microglia-mediated neuroinflammation.

### IC100 penetrates the brain and spinal cord

**Fig. 5:**
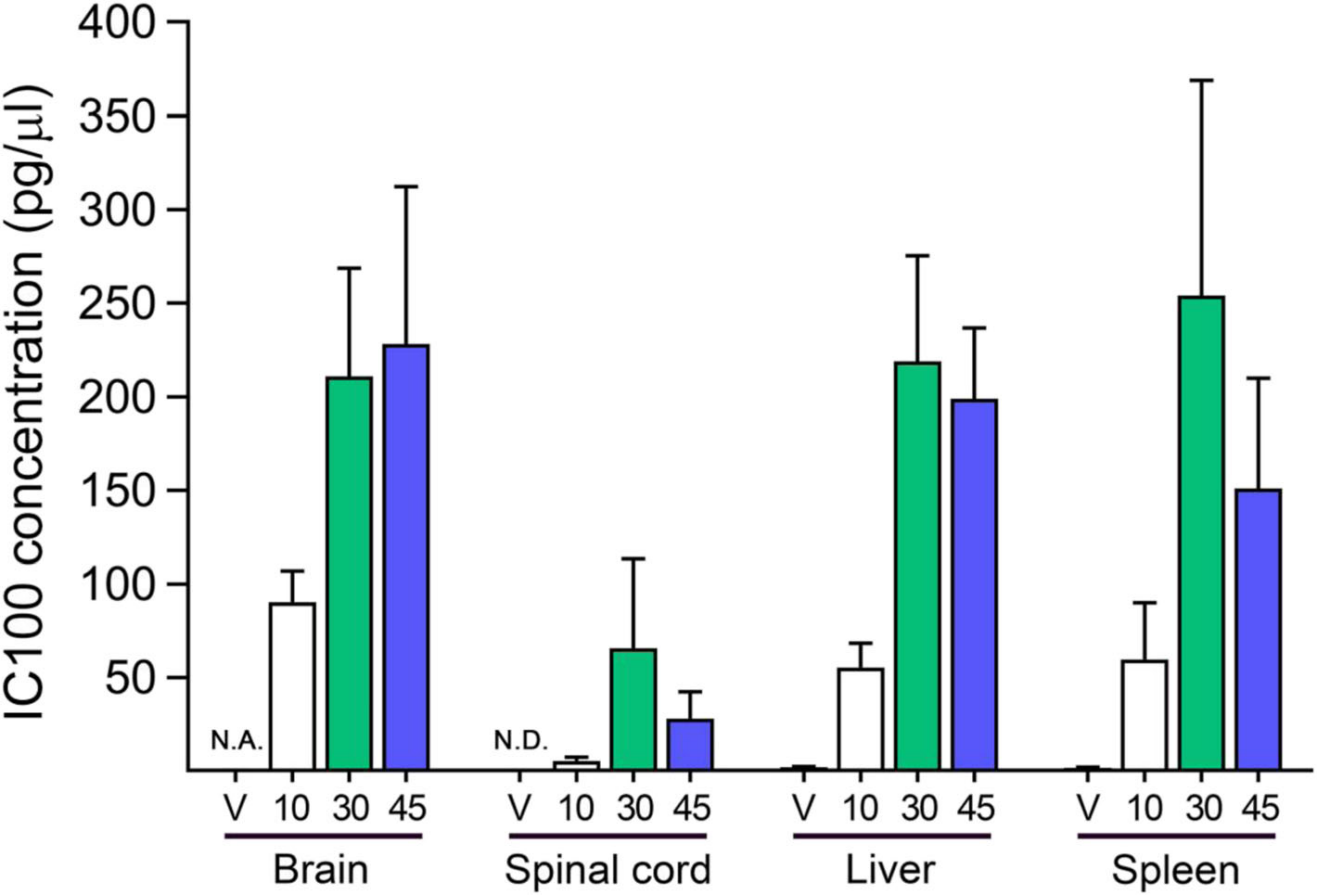
IC100 concentration in tissues. Concentrations of IC100 in pg/μl in the brain, spinal cord, liver, and spleen of mice treated with IC100 at 10, 30, and 45 mg/kg or vehicle at 35 dpi EAE. Results are expressed as mean ± SEM of 5–10 mice/group. N.A., not assessed; N.D., not detected

An important parameter in designing drugs to treat MS, particularly if they are meant to target neuroinflammation, is to determine whether they penetrate and accumulate in the CNS at therapeutic concentrations. This is especially important in the treatment of the progressive MS forms, where the blood-brain barrier appears relatively intact [31]. Thus, we quantified the levels of IC100 in the brain, spinal cord, liver, and spleen. IC100 penetrated the tissues analyzed at all three dosages (Fig. 5). The levels of IC100 in the spinal cord and brain were maximal at the 30 and 45 mg/kg doses, consistent with their therapeutic effect on reducing EAE severity (Fig. 1).

## Discussion

In this study, we investigated the functional and immunological effects of IC100, a monoclonal antibody against the inflammasome protein ASC, in the EAE animal model of MS. Here we show that ASC plays a critical role in the development of EAE, and that, by blocking ASC, the adaptive and innate immune response that sustain EAE are significantly reduced. Indeed, IC100 decreased the number of CD4^+^, CD8^+^ T, and CD11b^+^MHCII^+^ cells trafficking into the CNS, while reducing the number and activation state of CNS resident microglia. This is the first report to show that targeting ASC with a monoclonal antibody in MOG-induced EAE significantly alters innate and adaptive immune responses leading to improved clinical outcomes. Importantly, this work constitutes a novel approach to identify new drugs targeting the inflammasome that could be useful for the treatment of MS.

Our previous studies have shown that ASC is a reliable disease biomarker found in serum of patients with MS [9], traumatic brain injury [32, 33], and depression [25], as well as in extracellular vesicles isolated from individuals with stroke [11]. It is possible that IC100 works extracellularly to prevent ASC oligomerization into highly cross-linked macromolecular assemblies. On the other hand, ASC or ASC-IC100 complexes may be taken up by cells and internalized to prevent recruitment of ASC into the inflammasome complex. Previous studies in our laboratory have shown that anti-ASC is taken up by spinal cord neurons after trauma [10] and by the human monocytic leukemia cell line (THP-1 cells, unpublished data). It has been shown that monoclonal antibodies enter cells by receptor-mediated endocytosis through binding of the Fc domain to Fc-gamma-receptors (FcγRs) expressed on many immune cells, including monocytes, macrophages, microglia, myeloid progenitor cells, and dendritic cells [34, 35]. However, it is generally accepted that low affinity FcγRs differ from other receptors in that their activation requires cross-linking of polyvalent immune complexes, such as when multiple antibodies are bound to a protein aggregate [36, 37]. Multiple studies have suggested a role for microglia-mediated clearance of aggregated proteins as α–synuclein [38], Aß aggregates [31, 34], and Tau [15]. The later study also observed a clear size dependence of the microglial clearance pathway. Since ASC is secreted and oligomerizes into ASC specks, it is possible that IC100 may identify ASC assemblies and then are taken up by immune cells by receptor-mediated endocytosis. Therefore, establishing the precise specific mechanism of IC100 activity will help to design and optimize efficacy in vivo.

IL-1β/IL-18 secreted after inflammasome activation induces neutrophils and macrophages to engulf pathogens, promote inflammatory cell recruitment, and further amplify inflammatory responses [39, 40]. Apart from its impact on innate immunity, inflammasome activation also initiates adaptive immunity through the modulation of T helper subsets, skewing T cell differentiation in favor of Th1 and Th17 phenotypes [41, 42]. IL-1β also activates IL-1R signaling, which has a substantial influence on Th17 cell differentiation and related immune responses [43]. Several studies with ASC^−/−^ mice have suggested inflammasome-independent roles of ASC in the adaptive immune response. A novel microbial pathogen-induced mechanism for ASC in activating chemokine expression through MAPK activation, independent of the conventional caspase-1 inflammasome, has been reported [44]. Two studies of antigen-induced murine arthritis show dependence on ASC, but caspase-1, NLRP3, and NLRC4 independence [17]. A requirement for ASC, but not NLRP3 or caspase-1, was also demonstrated for antigen-specific humoral immunity after vaccination with MF59-adjuvented influenza [16]. In addition, ASC^−/−^ mice have reduced numbers of MOG-specific T cells in the lymph nodes and CNS, resulting in protection from EAE [19]. Lastly, genetic evidence shows an unexpected role of ASC in regulating motility of T lymphocytes and B lymphocytes and antigen uptake by professional antigen-presenting cells independently of inflammasomes [17]. In this study, ASC was shown to regulate the mRNA stability and expression of Dock2, a guanine nucleotide-exchange factor that mediates Rac-dependent signaling in immune cells [17].

The blood-brain barrier (BBB) presents a great impediment for brain drug delivery in that it is questioned whether sufficient intralesional drug concentrations can be reached after systemic administration. This problem is especially evident for biologicals such as monoclonal antibodies (mAbs) that are relatively large molecules. To overcome the presumed drug delivery obstacle in the brain, many treatment strategies for CNS diseases are directed at disrupting, passing, or bypassing the effective BBB [45, 46]. Disruption has been tried chemically, by drugs that influence passive diffusion (e.g., bradykinin, mannitol, regadenoson, and borneol) or active transport mechanisms (e.g., elacridar), or by radiotherapy, ultrasound, or microwaves. Moreover, via viral vectors, nanoparticles, liposomes, exosomes, and transporter or receptor ligands have been tried to bypass the BBB [45, 46].

However, the BBB is disrupted under various pathological conditions of diseases such as stroke, diabetes, seizures, hypertensive encephalopathy, acquired immunodeficiency syndrome, traumatic brain injuries, multiple sclerosis, Parkinson’s disease (PD), and Alzheimer disease (AD) [46]. It appears that in some pathological conditions, remodeling of the protein complex in interendothelial junctions is an important reason for the BBB breakdown [46]. Therefore, in CNS disease conditions associated with a leaky BBB, mAbs have been widely explored because of their specificity and high affinity for critical disease targets. Additionally, other important areas that deserve further investigation are the influence of aging [46] and major depressive disorder [47] on BBB dysfunctions and to discover whether mAbs may be used as possible approaches to deliver drugs into the brain to treat these conditions.

## Conclusions

Our data reveal that administration of IC100 (30 mg/kg) in MOG-induced EAE resulted in significant decrease in the number of CD4^+^, CD8^+^, and CD11b^+^MHCII^+^ cells in the spinal cord resulting in protection from EAE. In addition, this dose of IC100 also decreased the numbers of total and activated microglia in the spinal cord and resulted in improved clinical scores. These findings are consistent with our previous studies demonstrating that antibody-mediated neutralization of ASC results in significant functional improvements in the spinal cord [10] and brain trauma [48] injury models. Nevertheless, further studies are needed to uncover specific mechanisms of IC100 antibody activity and whether IC100 influences inflammasome-dependent and/or inflammasome-independent signaling cascades modulating adaptive and innate immune cell functions, and/or the trafficking of lymphocytes and myeloid cells into the CNS.

Decades of pharmacological research in EAE and MS have led to the development of mAbs against a variety of targets which have revolutionized MS treatment with an improvement in effectiveness. In the present study, we have discovered that IC100, which targets the inflammasome adaptor protein ASC, exhibits therapeutic properties in EAE and suppresses immune cell responses underlying tissue destruction. These findings provide a basis for the further development of anti-ASC drugs for the treatment not only of MS, but also for other neurodegenerative and traumatic CNS pathologies.

## Supplementary information

**Additional file 1:** Supplementary Table 1. Antibodies for flow cytometry.

**Additional file 2:** Supplementary Figure 1. Flow cytometry gating strategy. A representative spinal cord sample is shown.

## Data Availability

The data that support the findings are not publicly available. Data are available from the authors upon request.

## Abbreviations

ASC: Apoptosis-associated speck-like protein containing a caspase-recruitment domain
MS: Multiple sclerosis
EAE: Experimental autoimmune encephalomyelitis
MOG: Myelin oligodendrocyte glycoprotein
CNS: Central nervous system
RRMS: Relapsing-remitting
SPMS: Secondary-progressive
IL: Interleukin
DOCK-2: Dedicator of cytokinesis 2
NLRP: NOD-like receptor protein
NLRC4: NLR Family CARD Domain Containing 4
mAb: Monoclonal antibodies
FDA: Food and Drug Administration
CD: Cluster of differentiation
IgG: Immunoglobulin
HBSS w/o: Hanks’ Balanced Salt Solution without Mg2+ and Ca2+
RBC: Red blood cells
FCB: Flow cytometry buffer
SEM: Standard error of the mean
dpi: Days post-induction
CDI: Cumulative disease index
Th: T helper

## References

[1] Nylander A, Hafler DA (2012). Multiple sclerosis. J Clin Invest.

[2] Compston A, Coles A (2008). Multiple sclerosis. Lancet.

[3] Dobson R, Giovannoni G (2019). Multiple sclerosis - a review. Eur J Neurol.

[4] Constantinescu CS, Farooqi N, O'Brien K, Gran B (2011). Experimental autoimmune encephalomyelitis (EAE) as a model for multiple sclerosis (MS). Br J Pharmacol.

[5] de Alba E. Structure, interactions and self-assembly of ASC-dependent inflammasomes. Arch Biochem Biophys. 2019. 10.1016/j.abb.2019.05.023 PMC8455077 31152698

[6] Franklin BS, Bossaller L, De Nardo D, Ratter JM, Stutz A, Engels G (2014). The adaptor ASC has extracellular and ‘prionoid’ activities that propagate inflammation. Nat Immunol.

[7] Baroja-Mazo A, Martin-Sanchez F, Gomez AI, Martinez CM, Amores-Iniesta J, Compan V (2014). The NLRP3 inflammasome is released as a particulate danger signal that amplifies the inflammatory response. Nat Immunol.

[8] Franklin BS, Latz E, Schmidt FI (2018). The intra- and extracellular functions of ASC specks. Immunol Rev.

[9] Keane RW, Dietrich WD, de Rivero Vaccari JP (2018). Inflammasome proteins as biomarkers of multiple sclerosis. Front Neurol.

[10] de Rivero Vaccari JP, Lotocki G, Marcillo AE, Dietrich WD, Keane RW (2008). A molecular platform in neurons regulates inflammation after spinal cord injury. J Neurosci.

[11] Kerr N, Garcia-Contreras M, Abbassi S, Mejias NH, Desousa BR, Ricordi C (2018). Inflammasome proteins in serum and serum-derived extracellular vesicles as biomarkers of stroke. Front Mol Neurosci.

[12] Kerr NA, de Rivero Vaccari JP, Umland O, Bullock MR, Conner GE, Dietrich WD, et al. Human lung cell pyroptosis following traumatic brain injury. Cells. 2019;8. 10.3390/cells8010069 PMC6356886 30669285

[13] Lee SW, de Rivero Vaccari JP, Truettner JS, Dietrich WD, Keane RW (2019). The role of microglial inflammasome activation in pyroptotic cell death following penetrating traumatic brain injury. J Neuroinflammation.

[14] Lee SW, Gajavelli S, Spurlock MS, Andreoni C, de Rivero Vaccari JP, Bullock MR (2018). Microglial inflammasome activation in penetrating ballistic-like brain injury. J Neurotrauma.

[15] Funk KE, Mirbaha H, Jiang H, Holtzman DM, Diamond MI (2015). Distinct therapeutic mechanisms of tau antibodies: promoting microglial clearance versus blocking neuronal uptake. J Biol Chem.

[16] Ellebedy AH, Lupfer C, Ghoneim HE, DeBeauchamp J, Kanneganti TD, Webby RJ (2011). Inflammasome-independent role of the apoptosis-associated speck-like protein containing CARD (ASC) in the adjuvant effect of MF59. Proc Natl Acad Sci U S A.

[17] Ippagunta SK, Brand DD, Luo J, Boyd KL, Calabrese C, Stienstra R (2010). Inflammasome-independent role of apoptosis-associated speck-like protein containing a CARD (ASC) in T cell priming is critical for collagen-induced arthritis. J Biol Chem.

[18] Kolly L, Karababa M, Joosten LA, Narayan S, Salvi R, Petrilli V (2009). Inflammatory role of ASC in antigen-induced arthritis is independent of caspase-1, NALP-3, and IPAF. J Immunol.

[19] Shaw PJ, Lukens JR, Burns S, Chi H, McGargill MA, Kanneganti TD (2010). Cutting edge: critical role for PYCARD/ASC in the development of experimental autoimmune encephalomyelitis. J Immunol.

[20] Soares JL, Oliveira EM, Pontillo A (2019). Variants in NLRP3 and NLRC4 inflammasome associate with susceptibility and severity of multiple sclerosis. Mult Scler Relat Disord.

[21] Khan N, Kuo A, Brockman DA, Cooper MA, Smith MT (2018). Pharmacological inhibition of the NLRP3 inflammasome as a potential target for multiple sclerosis induced central neuropathic pain. Inflammopharmacology.

[22] Gris D, Ye Z, Iocca HA, Wen H, Craven RR, Gris P (2010). NLRP3 plays a critical role in the development of experimental autoimmune encephalomyelitis by mediating Th1 and Th17 responses. J Immunol.

[23] Inoue M, Williams KL, Gunn MD, Shinohara ML (2012). NLRP3 inflammasome induces chemotactic immune cell migration to the CNS in experimental autoimmune encephalomyelitis. Proc Natl Acad Sci U S A.

[24] Voge NV, Alvarez E. Monoclonal antibodies in multiple sclerosis: present and future. Biomedicines. 2019;7. 10.3390/biomedicines7010020 PMC6466331 30875812

[25] Syed YY (2018). Ocrelizumab: a review in multiple sclerosis. CNS Drugs.

[26] Chin P, Chan AC (2018). Ocrelizumab: a new therapeutic paradigm for multiple sclerosis: published as part of the biochemistry series “biochemistry to bedside”. Biochemistry.

[27] Mulero P, Midaglia L, Montalban X (2018). Ocrelizumab: a new milestone in multiple sclerosis therapy. Ther Adv Neurol Disord.

[28] Gao H, Danzi MC, Choi CS, Taherian M, Dalby-Hansen C, Ellman DG (2017). Opposing functions of microglial and macrophagic TNFR2 in the pathogenesis of experimental autoimmune encephalomyelitis. Cell Rep.

[29] Mejias NH, Martinez CC, Stephens ME, de Rivero Vaccari JP (2018). Contribution of the inflammasome to inflammaging. J Inflamm (Lond).

[30] Brambilla R, Morton PD, Ashbaugh JJ, Karmally S, Lambertsen KL, Bethea JR (2014). Astrocytes play a key role in EAE pathophysiology by orchestrating in the CNS the inflammatory response of resident and peripheral immune cells and by suppressing remyelination. Glia.

[31] Lassmann H, van Horssen J, Mahad D (2012). Progressive multiple sclerosis: pathology and pathogenesis. Nat Rev Neurol.

[32] Kerr N, Lee SW, Perez-Barcena J, Crespi C, Ibanez J, Bullock MR (2018). Inflammasome proteins as biomarkers of traumatic brain injury. PLoS One.

[33] Adamczak S, Dale G, de Rivero Vaccari JP, Bullock MR, Dietrich WD, Keane RW (2012). Inflammasome proteins in cerebrospinal fluid of brain-injured patients as biomarkers of functional outcome: clinical article. J Neurosurg.

[34] Gessner JE, Heiken H, Tamm A, Schmidt RE (1998). The IgG fc receptor family. Ann Hematol.

[35] Ryman JT, Meibohm B (2017). Pharmacokinetics of monoclonal antibodies. CPT Pharmacometrics Syst Pharmacol.

[36] Garcia-Garcia E, Rosales C (2002). Signal transduction during fc receptor-mediated phagocytosis. J Leukoc Biol.

[37] Metzger H, Kinet JP (1988). How antibodies work: focus on fc receptors. FASEB J.

[38] Bae EJ, Lee HJ, Rockenstein E, Ho DH, Park EB, Yang NY (2012). Antibody-aided clearance of extracellular alpha-synuclein prevents cell-to-cell aggregate transmission. J Neurosci.

[39] He Y, Hara H, Nunez G (2016). Mechanism and regulation of NLRP3 inflammasome activation. Trends Biochem Sci.

[40] van de Veerdonk FL, Netea MG, Dinarello CA, Joosten LA (2011). Inflammasome activation and IL-1beta and IL-18 processing during infection. Trends Immunol.

[41] Evavold CL, Kagan JC (2018). How inflammasomes inform adaptive immunity. J Mol Biol.

[42] Mills KH, Dungan LS, Jones SA, Harris J (2013). The role of inflammasome-derived IL-1 in driving IL-17 responses. J Leukoc Biol.

[43] Chung Y, Chang SH, Martinez GJ, Yang XO, Nurieva R, Kang HS (2009). Critical regulation of early Th17 cell differentiation by interleukin-1 signaling. Immunity.

[44] Taxman DJ, Holley-Guthrie EA, Huang MT, Moore CB, Bergstralh DT, Allen IC (2011). The NLR adaptor ASC/PYCARD regulates DUSP10, mitogen-activated protein kinase (MAPK), and chemokine induction independent of the inflammasome. J Biol Chem.

[45] Veldhuijzen van Zanten SEM, De Witt Hamer PC, van Dongen GAMS (2019). Brain access of monoclonal antibodies as imaged and quantified by ^89^Zr-antibody PET: perspectives for treatment of brain diseases. J Nucl Med.

[46] Dong X (2018). Current strategies for brain drug delivery. Theranostics.

[47] Cheung Y, Desse S, Martinez A, Worthen RJ, Jope RS, Beurel E (2018). TNFα disrupts blood brain barrier integrity to maintain prolonged depressive-like behavior in mice. Brain Behav Immun.

[48] de Rivero Vaccari JP, Lotocki G, Bramlett HM, Dietrich WD, Keane RW, Alonso OF (2009). Therapeutic neutralization of the NLRP1 inflammasome reduces the innate immune response and improves histopathology after traumatic brain injury. J Cereb Blood Flow Metab.

