# Supplementary material for: IC100: a novel anti-ASC monoclonal antibody improves functional outcomes in an animal model of multiple sclerosis
Source: J Neuroinflammation. 2020 May 4;17:143. doi: 10.1186/s12974-020-01826-0 (PMC7199312; doi:10.1186/s12974-020-01826-0)
Supplement: Supplementary file 1 — Additional file 1: Supplementary Table 1. Antibodies for flow cytometry. [file 12974_2020_1826_MOESM1_ESM.docx]

**Supplementary Table 1.** Antibodies for flow cytometry.

| **Antigen** | **Color** | **Dilution** | **Provider** | **Catalog #** |
| --- | --- | --- | --- | --- |
| CD45 | FITC | 1:1000 | eBioscience | 11-0451-82 |
| CD4 | PE/Cy7 | 1:200 | eBioscience | 25-0042-81 |
| CD8 | Percp-Cy 5.5 | 1:200 | Biolegend | 100734 |
| B220 | PE | 1:200 | Biolegend | 103208 |
| CD11b | APC eFluor780 | 1:200 | eBioscience | 47-0112-82 |
| MHCII | APC | 1:200 | eBioscience | 17-5321-81 |
