# Supplementary figures and images for: IC100: a novel anti-ASC monoclonal antibody improves functional outcomes in an animal model of multiple sclerosis
Source: J Neuroinflammation. 2020 May 4;17:143. doi: 10.1186/s12974-020-01826-0 (PMC7199312; doi:10.1186/s12974-020-01826-0)

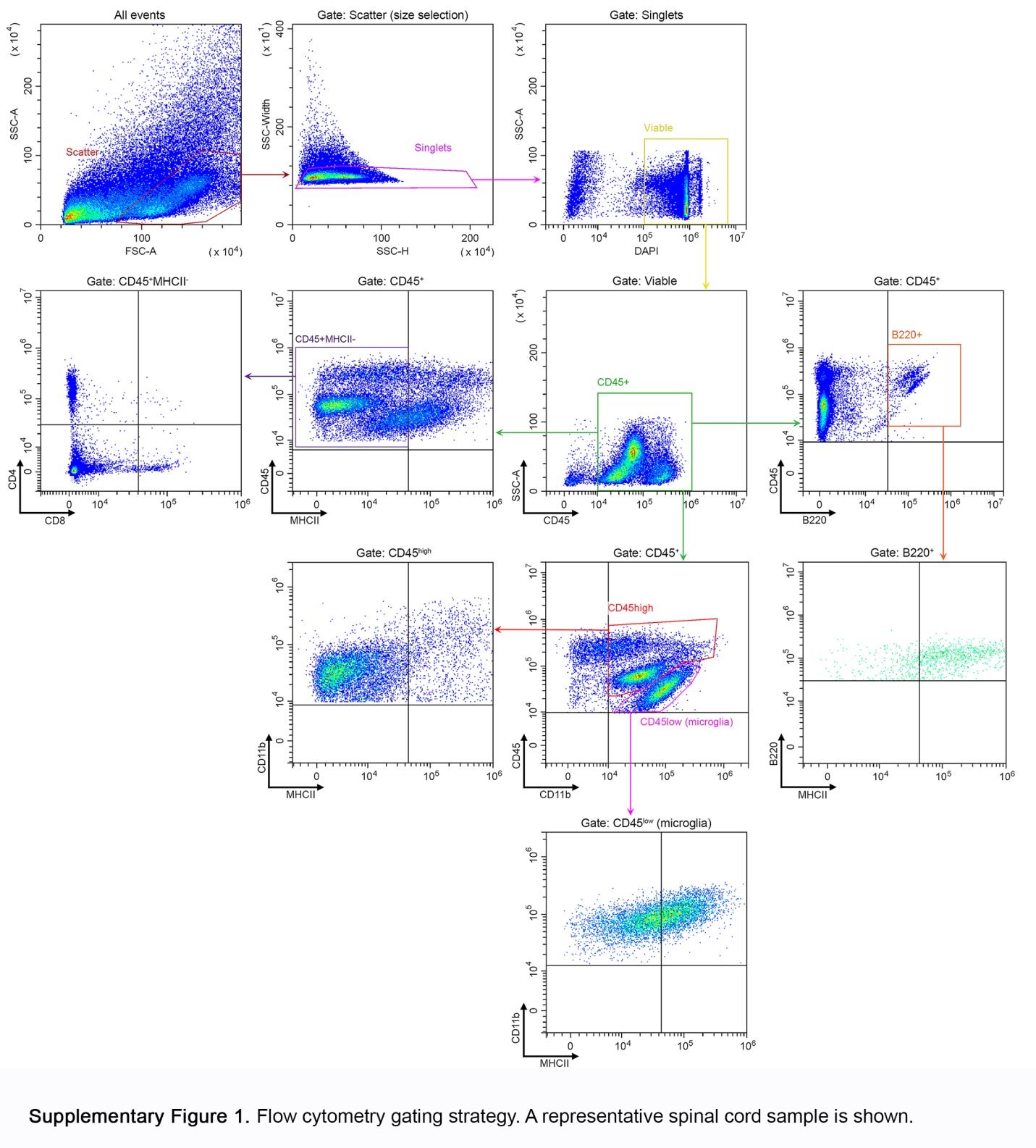

Supplement: Supplementary file 2 — Additional file 2: Supplementary Figure 1. Flow cytometry gating strategy. A representative spinal cord sample is shown. [file 12974_2020_1826_MOESM2_ESM.docx]
